# Synergistic effects of 13-year warming and nitrogen fertilization accelerating soil carbon destabilization in North China Plain farmland

**DOI:** 10.3389/fmicb.2026.1775179

**Published:** 2026-04-16

**Authors:** Chuang Zhang, Wenxu Dong, Jing Wang, Wenyan Li, Ruiyuan Zhang, Xiaoxin Li, Xiuping Liu, Yang Yang, Yuming Zhang, Chunsheng Hu

**Affiliations:** 1 Key Laboratory of Remote Sensing and Geographic Information Systems in Henan Province, Institute of Geographical Sciences, Henan Academy of Sciences, Zhengzhou, China; 2 Hebei Laboratory of Soil Ecology, Center for Agricultural Resources Research, Institute of Genetic and Developmental Biology, Chinese Academy of Sciences, Shijiazhuang, China; 3 University of Chinese Academy of Sciences, Beijing, China; 4 Department of Municipal and Environmental Engineering, Hebei University of Architecture, Zhangjiakou, China

**Keywords:** global warming, isotopic tracing, microbial metabolism, nitrogen additions, North China Plain farmland, soil carbon cycling

## Abstract

Soil carbon stabilization is critical for mitigating carbon decomposition under global change scenarios. However, the long-term combined effects of warming and nitrogen fertilization on the stabilization of the soil carbon pool in farmland remain poorly understood. Through a 13-year field experiment conducted in the North China Plain, we investigated the response of soil carbon pool components under four treatments: Ambient control (CK), warming (W_1.5 °C_), nitrogen fertilization (N_240_), and combined warming with nitrogen fertilization (WN). A three-pool carbon decomposition model (active, slow, and passive carbon pools) was employed to characterize carbon stabilization. Soils with straw addition were incubated indoors to quantify priming effects (PEs), complemented by microbial biomass carbon (MBC) measurements and δ^13^C isotopic tracing. Our results revealed that the WN treatment reduced passive carbon by 26%, exceeding the effects of individual treatments (10–18% reduction under warming and 6–8% under nitrogen fertilization). However, the slow carbon pool size increased by 41% under the WN treatment, surpassing the effects of individual treatments (18% under warming and 7–9% under nitrogen fertilization). These findings suggest that the combined treatment accelerated the decomposition of passive carbon pools and contributed to the accumulation of slow carbon pools, relative to single warming or nitrogen fertilization treatments. In soils amended with straw and incubated for 120 days, the WN treatment exhibited 42% higher MBC and 3.9‰ more depleted δ^13^C-MBC values compared to single-factor treatments and the CK. The WN treatment significantly reduced the cumulative PE by 3%, whereas single treatments increased it by 2–4%. Structural equation modeling identified soil total nitrogen (TN) and passive carbon pool size as the primary regulators of these contrasting effects. Our findings suggest that the long-term combination of warming and nitrogen fertilization could exacerbate soil carbon destabilization in farmland. Microbial metabolic shifts potentially serve as an important regulatory mechanism influencing agricultural soil carbon stocks under future climate warming.

## 1 Introduction

The global warming trajectory, with temperatures rising by approximately 1.5 °C above pre-industrial levels (IPCC, 2022; Matthews and Wynes, 2022; Deng et al., 2023), has caused substantial carbon losses (Crowther et al., 2016; Hicks et al., 2017). As the largest terrestrial carbon reservoir, soils store 2–3 times more carbon than the atmosphere and vegetation combined (Ciais et al., 2013), rendering even marginal perturbations in soil carbon pools pivotal to climate feedbacks. Although prolonged nitrogen (N) fertilization has enhanced global soil carbon sequestration (Chen et al., 2018b; Ye et al., 2018), it has also sparked debate about its capacity to offset warming-driven carbon losses (Sáez-Sandino et al., 2024). Soil carbon stabilization, defined as the ratio of passive to total organic carbon pools, critically governs carbon stocks. Microbial carbon use efficiency (CUE), the fraction of assimilated carbon allocated to biomass production versus respiratory losses, serves as a key determinant of soil carbon stabilization (Kuzyakov, 2010; Tao et al., 2023; Wang and Kuzyakov, 2024; Zhou et al., 2025). However, it remains unclear how the interplay of multiple climatic factors will reshape soil carbon pool components over prolonged temporal scales.

Chronic depletion of labile carbon coupled with intensifying nutrient stoichiometric imbalance under long-term warming may fundamentally alter soil carbon stabilization trajectories. For example, the inherent thermal sensitivity of active carbon typically enhances passive carbon accumulation (Bradford, 2013; Wang et al., 2014; Sáez-Sandino et al., 2023), thereby elevating microbial CUE and reinforcing soil carbon stabilization. However, emerging evidence from multi-decadal warming experiments reveals that microbial communities are progressively dominated by ligninolytic taxa (e.g., Basidiomycota) (Garcia-Palacios et al., 2015; Pold et al., 2015; Cheng et al., 2017; Metcalfe, 2017; Romero-Olivares et al., 2017), concomitant with global increases in oxidative-to-hydrolytic enzyme activity ratios (Chen et al., 2018a; Chen et al., 2020). This aligns with microbial strategies to overcome nutritional deficits, preferentially targeting passive carbon substrates and ultimately accelerating the decomposition of complex carbon.

Soil carbon stabilization under warming is further modulated by abiotic factors, such as precipitation and agricultural tillage practices. For instance, conventional tillage under four-year warming disrupts macroaggregates, mobilizing previously protected carbon (Hou et al., 2016). In contrast, warming-induced drought shows limited carbon impacts in semi-arid grasslands (Allison and Treseder, 2008; Niu et al., 2008). These divergent responses underscore the critical role of agricultural practices in mediating warming effects, necessitating further research to unravel biotic and abiotic factors governing soil carbon pool components in semi-arid farmland soils.

Warming-induced nutrient stoichiometric imbalances—that is, N and phosphorus (P) limitation—critically regulate soil carbon stabilization (Bai et al., 2023; Shi et al., 2023). For example, according to nitrogen mining theory, microbial N demand drives the decomposition of organic carbon (Averill and Waring, 2018). Accelerated N mineralization under warming reduces soil C:N ratios (Bai et al., 2023). Our previous eight-year warming experiment showed a marked decline in total nitrogen (TN) content (Zhang C. et al., 2021). Despite the long-term combined effects of warming and N fertilization increasing soil carbon emissions more than warming alone (Zhou et al., 2016), their combined effects on the decomposition of soil carbon varied across contrasting ecosystems. For example, no combined effects on enzyme activities were found in temperate grassland soils (Thakur et al., 2019). Positive combined effects on C-, N-, and P-acquiring hydrolases activities were observed in North China Plain farmland soils (Zhang C. et al., 2021). In temperate forests and grasslands, soils exposed to 4 years of warming exhibited greater P limitation compared to N (Gong et al., 2020; Shi et al., 2023). In addition, artificial irrigation offset their positive combined effects on microbial community biomass and diversity, which aim to decompose organic carbon (Zhu et al., 2021). These findings imply that the direction of combined effects between warming and nitrogen is controlled by abiotic factors and microbial nutrient demands. It therefore remains challenging to elucidate how N fertilization in farmland affects the stabilization of soil carbon pools under climate warming.

The North China Plain annually produces a substantial straw biomass of 2.8 Gt (Liu et al., 2014; Zhang X. et al., 2021). Straw incorporation triggers priming effects (PEs), accelerating the decomposition of native soil organic carbon (SOC) (Chen et al., 2014; Bastida et al., 2019; Liu et al., 2020; Li et al., 2023), mediated by microbial resource allocation trade-offs. Active carbon pool size has emerged as a pivotal determinant of the magnitude and direction of PE (Liu et al., 2017; Liu et al., 2020), with larger active carbon pools in soils exhibiting higher PEs under chronic warming (Mau et al., 2018). Low-CUE communities enhanced PEs in soils exposed to long-term warming, and the reverse was also observed (Mau et al., 2018). In soils exposed to long-term warming, straw incorporation accelerated the decomposition of passive carbon (Guenet et al., 2012; Chen et al., 2022; Zhang et al., 2023), ultimately augmenting PEs (Gunina and Kuzyakov, 2022). Warming has elicited variable responses in PEs caused by straw addition, with decreases observed in farmland (Li et al., 2023), no significant change in forest (Guttières et al., 2021), and increases in grassland (Tao et al., 2024). In addition, variance in the soil C:N ratio, pH, and aridity modulates PEs in different directions. Warming intensified PEs caused by straw addition in soils with high initial C:N ratios (Li et al., 2023) but mitigated PEs in alkaline soils (Wang and Kuzyakov, 2024). Warming increased PEs caused by straw addition under tillage, in contrast to the minimal responses observed in no-till soils (Li et al., 2023). These findings underscore a critical knowledge gap: How long-term warming combined with N fertilization affects both carbon stabilization and microbial CUE, ultimately influencing the PE trajectory in croplands.

Our research aims to elucidate how the combined long-term effects of warming and nitrogen fertilization reshape soil carbon stabilization. Due to the preferential utilization of active carbon, we hypothesize that (1) long-term warming alone will relatively accumulate passive carbon, thereby consolidating the stabilization of soil carbon pools. (2) Nitrogen additions, by compensating microbial nitrogen deficits, in combination with long-term warming, will facilitate the degradation of passive carbon pools.

## 2 Materials and methods

### 2.1 Study site and experimental design

Our study was conducted at the Luancheng Ecological Station of the Chinese Academy of Sciences in Hebei Province, China (37°53′N, 114°41′E, 50 m above sea level). The site, an integral part of the North China Plain, represents farmland that has followed a winter wheat–maize/soybean rotation cropping system for decades. The region exhibits a distinct semi-arid climate influenced by monsoon patterns, with a mean annual temperature of 12 °C and an average annual precipitation of 460 mm. The soil profile is characterized by a sandy loam texture, comprising 54% sand, 34% silt, and 12% clay, and is classified as Hapli-Ustic Inceptisols according to the US soil taxonomy system. The topsoil layer (0–20 centimeters in depth) contains organic matter of 15 g kg^−1^ and total nitrogen (TN) of 1 g kg^−1^, with a bulk density of 1.3 g cm^−3^ and a pH of 8.0 (Liu et al., 2013; Li et al., 2019).

The long-term warming and N fertilization experiment started in October 2009 using a completely randomized experimental design. The study included four distinct treatments, each replicated three times across a total of 12 plots, with each plot measuring 4 m × 4 m. These treatments included an untreated control (CK), which remained unexposed to either warming or N fertilization. For the N fertilization (N_240_) and combined warming plus N (WN) treatments, urea was uniformly applied at a rate of 240 kg^−1^ N ha^−1^ year^−1^, distributed across three time points: November (50%), March (25%), and May (25%). This application schedule was designed to optimize nutrient availability throughout the growing season. Regarding the warming (W_1.5 °C_) and WN treatments, warming was applied to the central 2 m × 2 m area of each 4 m × 4 m plot. A total of three 2-m infrared heating tubes, each with a rated power of 1,000 W, were installed at equal intervals and suspended 1.5 m above the ground, creating a concentrated effective radiation area of 2 m × 2 m. This arrangement ensured continuous and uniform heating for 24 h per day within the designated plots. Over the past decade, this heating regimen has led to a notable increase in the annual average soil temperature at a depth of 10 cm by approximately 1.5 °C. To minimize potential confounding factors, all 12 plots were equipped with identical warming infrastructure. However, a deliberate power cutoff was implemented for the six plots assigned to the CK and N fertilization treatments.

All 12 experimental plots followed a monoculture winter wheat system. Winter wheat was sown in late October and harvested in early June. After harvest, the straw was removed from all plots. Then, the plots entered a fallow period from June to October, completing the annual crop-fallow cycle. Prior to sowing, Ca(H_2_PO_4_)_2_ was applied uniformly across all 12 plots at a rate of 65 kg P ha^−1^ year^−1^. The soil was then artificially tilled to a depth of 40 cm, ensuring adequate mixing of the applied fertilizer. Furthermore, 60 mm of tap water was evenly applied via flooding in March and May, targeting key growth stages sensitive to soil moisture.

### 2.2 Soil sampling and analyses

Soil samples were collected from the top 10 cm on 1 May 2022, marking the 13th year of the experiment. Sampling occurred approximately 1 month after the last irrigation and N fertilization events, ensuring that the soil microbial community had stabilized. Given that microbial biomass peaks in May (Zhang C. et al., 2021), this timing was ideal for conducting indoor incubation experiments. To eliminate marginal effects, soils were collected from a designated 2 m x 2 m area within the effective radiation zone of each plot. These samples were then transported to the laboratory in an insulated incubator. The samples were sieved through a 2 mm mesh to homogenize the soil and remove debris, followed by storage at 4 °C until further analysis. Soil gravimetric water content and ammonium concentrations were measured within 24 h of sample collection. Within 1 week of collection, the indoor incubation experiment was started.

Soil pH was measured using a digital meter, with a soil-to-water ratio of 10 g fresh soil to 25 mL CO_2_-free distilled water. Soil gravimetric water content was determined by oven-drying the soil samples at 105 °C for 24 h, allowing quantification of the soil respiration rate and weight-based water loss. Soil volumetric water content (SVWC) and temperature (soil T) at a depth of 10 cm were continuously monitored using a time-domain reflectometer (TDR 100 system, Campbell, USA), with readings taken hourly. For subsequent statistical analysis, the average SVWC and soil T were calculated over two-week periods immediately before and after the sampling event.

SOC and total nitrogen (TN) contents were accurately determined using an elemental analyzer (Vario MACRO cube, Elementar, Germany). Prior to analysis, carbonates and hydrocarbonates were removed from the soil samples through treatment with 1 mol L^−1^ HCl. Soil samples were then extracted with 0.5 mol L^−1^ KCl at a ratio of 10 g soil to 50 mL KCl. The resulting filtrate was analyzed for ammonium (NH_4_^+^) using a flow analyzer (SMARTCHEM 140, Italy), while nitrate (NO_3_^−^) concentrations were measured spectrophotometrically (UV-2450, Shimadzu, Japan) at wavelengths of 220 nm and 275 nm. Furthermore, 10 g of fresh soil was thoroughly mixed with 50 mL of 0.5 mol L^−1^ K_2_SO_4_. Dissolved organic carbon (DOC) content was determined using a flow analyzer (Vario TOC select, Elementar, Germany). In addition, HCl-treated soil samples and freeze-dried filtrates from the K_2_SO_4_ extraction were utilized to measure δ^13^C values of SOC and DOC, respectively, via an isotope mass spectrometer (Thermo Fisher Delta V PLUS). Soil available phosphorus (AP) was extracted with 0.5 mol L^−1^ NaHCO_3_ (pH 8.5), and phosphomolybdenum blue complexes were formed by reaction with a mixed reagent containing ammonium molybdate, antimony potassium tartrate, and ascorbic acid under acidic conditions. Then, the spectrophotometric value was quantified using continuous flow analysis (Auto Analyzer 3, Bran Luebbe, Germany).

### 2.3 Indoor incubation experiment

Fresh soils, equivalent to 10 g of air-dried soil, were adjusted to 60% of field water capacity and pre-incubated in black polyvinylidene fluoride gas bags with a maximum capacity of 500 mL for 3 days at 25 °C in the dark. Prior to incubation, each bag of the 10 parallel soil samples was injected with 300 mL of ambient air, and an additional five gas bags were filled solely with 300 mL of air to establish baseline CO_2_ concentrations and δ^13^C values. Incubation of unamended soil served as a blank and was used to fit a three-carbon-pool model based on first-order kinetics. To evaluate the PE, 0.33 mg of winter wheat straw (δ^13^C value = −27‰) was added to the soil samples with a background δ^13^C value of approximately −22‰, yielding an isotopic difference of approximately 5‰. Both straw-amended and straw-free soils were incubated simultaneously. Then, gas samples (100 mL) were collected from soil-only and straw-amended incubation at precise intervals: 2 h, 4 h, 6 h, 12 h, 1 day, 3 days, 5 days, 7 days, 14 days, 30 days, 45 days, 60 days, 75 days, 90 days, 105 days, and 120 days. After gas sampling, distilled water was added to maintain constant moisture levels according to the weight loss of water. Gas bags were opened for 30 min and then evacuated three times using a glass syringe. Subsequently, 300 mL of fresh air was injected into each bag to restore ambient O_2_ levels, preparing the system for the next sampling interval. Soil-only and straw-amended incubations were separately replicated four times, ensuring the collection of incubated soils at 3 days, 14 days, 30 days, and 120 days.

CO_2_ concentrations in the collected gas samples were measured using gas chromatography (Agilent 6820, Agilent, USA). The chloroform fumigation-extraction technique was employed to assess microbial biomass carbon (MBC) and its δ^13^C values. Briefly, 10 g of incubated soil samples were subjected to chloroform fumigation, followed by extraction with 0.5 mol L^−1^ potassium sulfate (K_2_SO_4_) solution. The resulting filtrate was then analyzed using a flow analyzer (Vario TOC select, Elementar, Germany). MBC was calculated as the difference in carbon content between fumigated and non-fumigated soil samples. The filtrate was then subjected to vacuum freeze-drying, ensuring adequate collection of the precipitates. δ^13^C values of CO_2_ and MBC were measured using an Isotope Ratio Mass Spectrometer (Thermo Fisher Delta V PLUS) and reported relative to the Vienna Peedee Belemnite (VPDB) standard.

### 2.4 Calculations and estimation of soil carbon pools

Respired CO_2_ (C_respired_) was calculated as the difference between CO_2_ concentration in the incubated sample (C_incubated_) and air CO_2_ (C_air_). The δ^13^C value of respired CO_2_ (δ^13^C_respired_) was determined from the incubated CO_2_ using the following Equation 1:


$$\delta^{13}C_{\text{respired}}=\frac{\delta^{13}C_{\mathrm{air}}\times C_{\mathrm{air}}-\delta^{13}C_{\text{incubated}}\times C_{\text{incubated}}}{C_{\text{incubated}}-C_{\mathrm{air}}}\;$$ (1)

δ^13^C_incubated_ and δ^13^C_air_ represent the corresponding δ^13^C value of CO_2_ in the headspace of the incubation bags and in ambient air, respectively.

The soil-derived respired CO_2_ (R_soil_) in the straw-amended incubation was calculated using the following Equation 2:


$$R_{\text{soil}}=C_{\text{respired}}\times \frac{\delta^{13}C_{\text{respired}}-\delta^{13}C_{\text{soil}}}{\delta^{13}\text{Csoil}-\delta^{13}\text{Cstraw}}$$ (2)

Here, δ^13^C_straw_ and δ^13^C_soil_ denote the δ^13^C values of the straw and soil, respectively.

The PE induced by straw was quantified as the difference in soil-derived CO_2_ concentrations between the straw-amended and soil-only incubations. Microbial carbon use efficiency (CUE), which reflects microbial carbon allocation to growth versus respiration (Tao et al., 2023), was calculated using the following Equation 3:


$$\mathrm{CUE}=\frac{\Delta \mathrm{MBC}}{\Delta \mathrm{MBC}+\Delta C_{\text{respired}}}$$ (3)

Following Jiang et al. (2018), the sizes and turnover rates of active, slow, and passive soil organic carbon pools were quantified using a three-carbon-pool model based on first-order kinetics Equation 4:


$$C_{t}=C_{a}\times e^{-\mathrm{Ka}*t}+C_{s}\times e^{-\mathrm{Ks}*t}+C_{p}\times e^{-\mathrm{Kp}*t}$$ (4)

**Figure 1:**
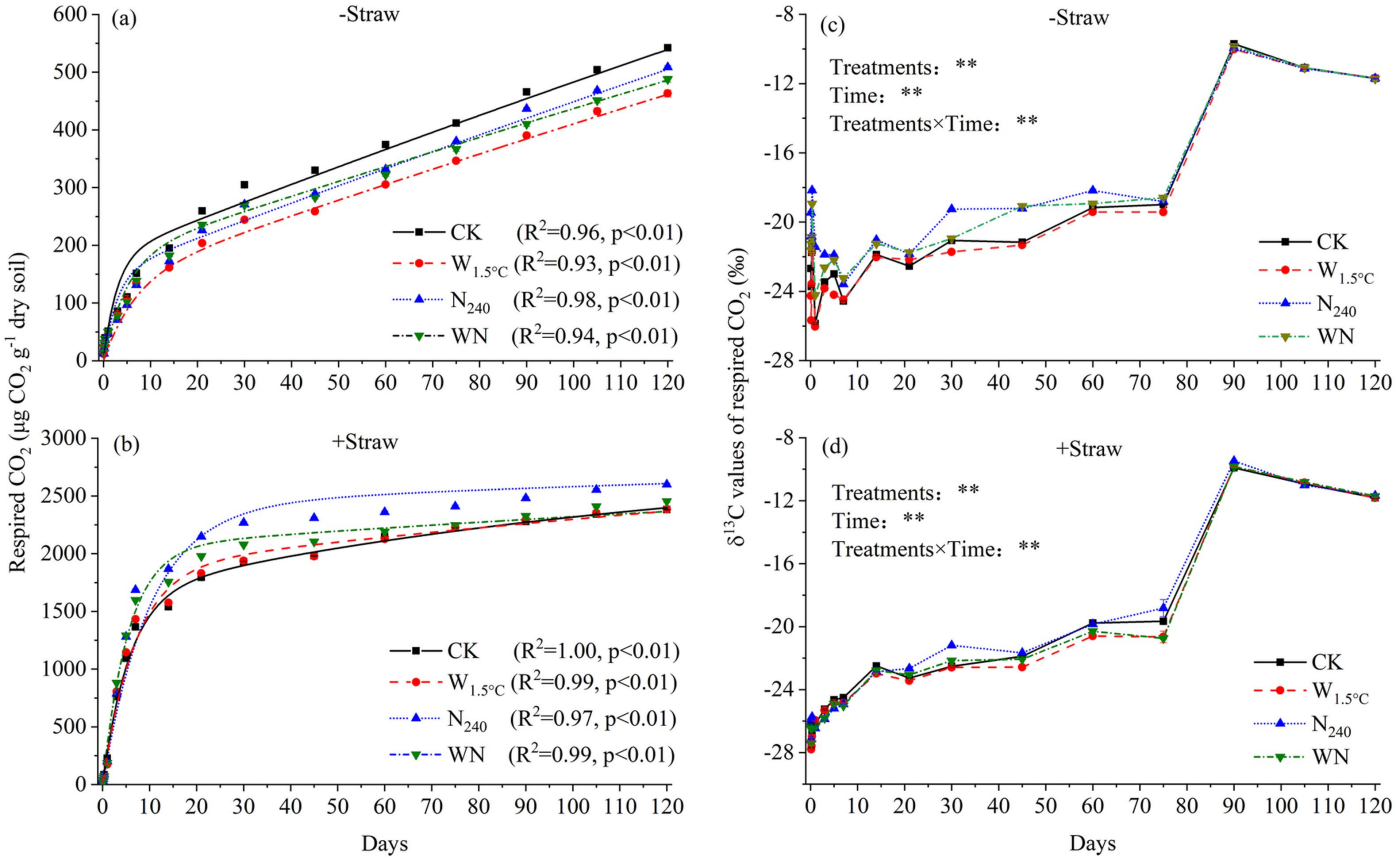
Dynamics of respired CO_2_ and δ^13^C-CO_2_ values in soils subjected to 15 years of warming and nitrogen fertilization, with air **(a,c)** and straw **(b,d)** amendments. Curved lines represent fits to a three-carbon-pool model based on a first-order kinetic equation.

Here, C_t_ represents the SOC content at incubation time t. C_a_, C_s_, and C_p_ denote the sizes of the active, slow, and passive carbon pools, respectively. K_a_, K_s,_ and K_p_ are the decomposition rate constants (reciprocals of mean residence time, 1/MRT) for the active, slow, and passive carbon pools. C_p_ was determined using the acid hydrolysis method. Specifically, 5 g of air-dried soil was hydrolyzed with 50 mL of 6 mol L^−1^ HCl for 18 h at 116 °C. The remaining SOC in the residual soil was defined as C_p_. K_p_ was set as a constant at 4.4 × 10^−6^ d^−1^, assuming an MRT_p_ of 620 years at an incubation temperature of 25 °C (Paul et al., 2001). C_s_ was calculated as C_s_ = C_t_ – C_a_ – C_p_. The values of K_a_ and K_s_ were estimated by fitting to the three-carbon-pool model (Figure 1).

### 2.5 Statistical analyses

One-way ANOVA followed by Tukey’s *post hoc* test was employed to assess significant differences among treatments in soil physical and chemical properties, carbon pools sizes and their percentage components, and PE, CUE, MBC, and δ^13^C-MBC during the incubation period. Two-Way ANOVA was used to evaluate the interactive effects of warming and N fertilization, as well as the differences between incubation times and treatments. The Mantel test was utilized to examine the relationship between MBC, PE, and CUE during incubation and soil physical and chemical properties. A piecewise structural equation modeling (pSEM) approach was employed to dissect the direct and indirect effects of carbon pool components, initial soil nutrient content (TN), and microbial properties (MBC and CUE) on cumulative PEs at specific incubation time points: 2 h, 3 days, and 30 days. In the pSEM analysis, treatments were included as a random factor within linear mixed-effects models. The adequacy of the pSEM was evaluated using Fisher’s C statistic in the “piecewiseSEM” package in R version 4.4.0. All statistical analyses and data visualization were performed using IBM SPSS Statistics version 19, OriginLab 2018, and R version 4.4.0.

## 3 Results

### 3.1 Soil physical and chemical properties in response to long-term warming and N fertilization

A total of 13 years of warming, compared to the control, increased soil temperature at a depth of 10 cm by approximately 2.4 °C and concomitantly caused a 3.7% decrease in SVWC. Similarly, an increase in soil T of 0.8 °C in the warming plus nitrogen treatment was associated with a significant 2.8% reduction in SVWC (Table 1). Long-term warming alone decreased TN content by approximately 9% and AP content by 29%, relative to the CK. However, warming alone increased NO_3_^−^ content by approximately 16% and DOC content by approximately 29%. Both N fertilization and WN treatments elevated NO_3_^−^ content by 12–13 times compared to the CK. In contrast, AP content declined by 15–24% in response to N fertilization, either alone or in combination with warming, whereas DOC content increased by 29–39% relative to the CK (Table 1).

**Table 1:** Soil physical and chemical properties in response to long-term warming and nitrogen fertilization, and the interactive effects of both treatments.

Treatments	SOC	δ^13^C-SOC	δ^13^C-C_p_	TN	SOC/TN	SVWC	pH	Soil T	NO_3_^−^	NH_4_^+^	AP	DOC	δ^13^C-DOC
	g kg^−1^	‰	‰	g kg^−1^		%		°C	mg kg^−1^	mg kg^−1^	mg kg^−1^	mg kg^−1^	‰
CK	7.7 ± 0.1 a	−23.21 ± 0.09 ab	−24.04 ± 0.20 a	0.98 ± 0.01 a	7.84 ± 0.05 ab	16.0 ± 0.2 a	7.9 ± 0.0 a	13.4 ± 0.1 c	1.9 ± 0.0 c	0.8 ± 0.0 a	55.5 ± 1.9 a	14.6 ± 0.2 b	−17.60 ± 0.07 b
W_1.5°C_	7.3 ± 0.1 b	−22.97 ± 0.12 a	−24.25 ± 0.17 a	0.89 ± 0.00 b	8.17 ± 0.14 a	12.3 ± 0.1 c	7.8 ± 0.0 a	15.8 ± 0.1 a	2.2 ± 0.1 c	0.6 ± 0.0 b	39.3 ± 1.2 c	18.8 ± 0.5 a	−17.72 ± 0.08 b
N_240_	7.7 ± 0.1 ab	−22.96 ± 0.11 a	−23.95 ± 0.07 a	1.02 ± 0.01 a	7.51 ± 0.14 b	16.0 ± 0.0 a	7.5 ± 0.0 b	13.1 ± 0.1 c	26.5 ± 0.9 a	0.6 ± 0.0 b	42.3 ± 0.3 bc	20.3 ± 0.4 a	−16.76 ± 0.18 a
WN	7.6 ± 0.0 ab	−23.50 ± 0.06 b	−24.31 ± 0.12 a	0.99 ± 0.02 a	7.67 ± 0.10 ab	13.2 ± 0.1 b	7.5 ± 0.0 b	14.2 ± 0.1 b	24.3 ± 0.3 b	0.8 ± 0.0 a	46.9 ± 1.0 b	18.8 ± 0.3 a	−17.53 ± 0.11 b
W_1.5°C_	ns	ns	ns	**	**	**	**	**	**	ns	**	**	**
N_240_	*	ns	ns	**	*	**	ns	**	ns	ns	**	**	**
W_1.5°C_ × N_240_	ns	**	ns	*	ns	**	ns	**	*	**	**	**	**

The reported values are presented as means ± standard errors (SE). Lowercase letters indicate significant differences between treatments (*n* = 3, *p* < 0.05). SOC, TN, and SOC/TN represent soil organic carbon, total nitrogen content, and their ratio, respectively. SVWC, soil T, and pH denote soil volumetric water content, temperature, and pondus hydrogenii at a depth of 10 cm. NO_3_^−^, NH_4_^+^, AP, and DOC denote soil nitrate, ammonium, available phosphorus, and dissolved organic carbon content, respectively. δ^13^C-SOC, δ^13^C-C_p_, and δ^13^C-DOC correspond to the δ^13^C values of SOC, passive carbon, and DOC, respectively. Asterisks (*) and (**) denote significance at *p* < 0.05 and *p* < 0.01, respectively. While ns denotes no significance (*p* > 0.05).

### 3.2 Soil carbon pools in response to long-term warming and N fertilization

**Figure 2:**
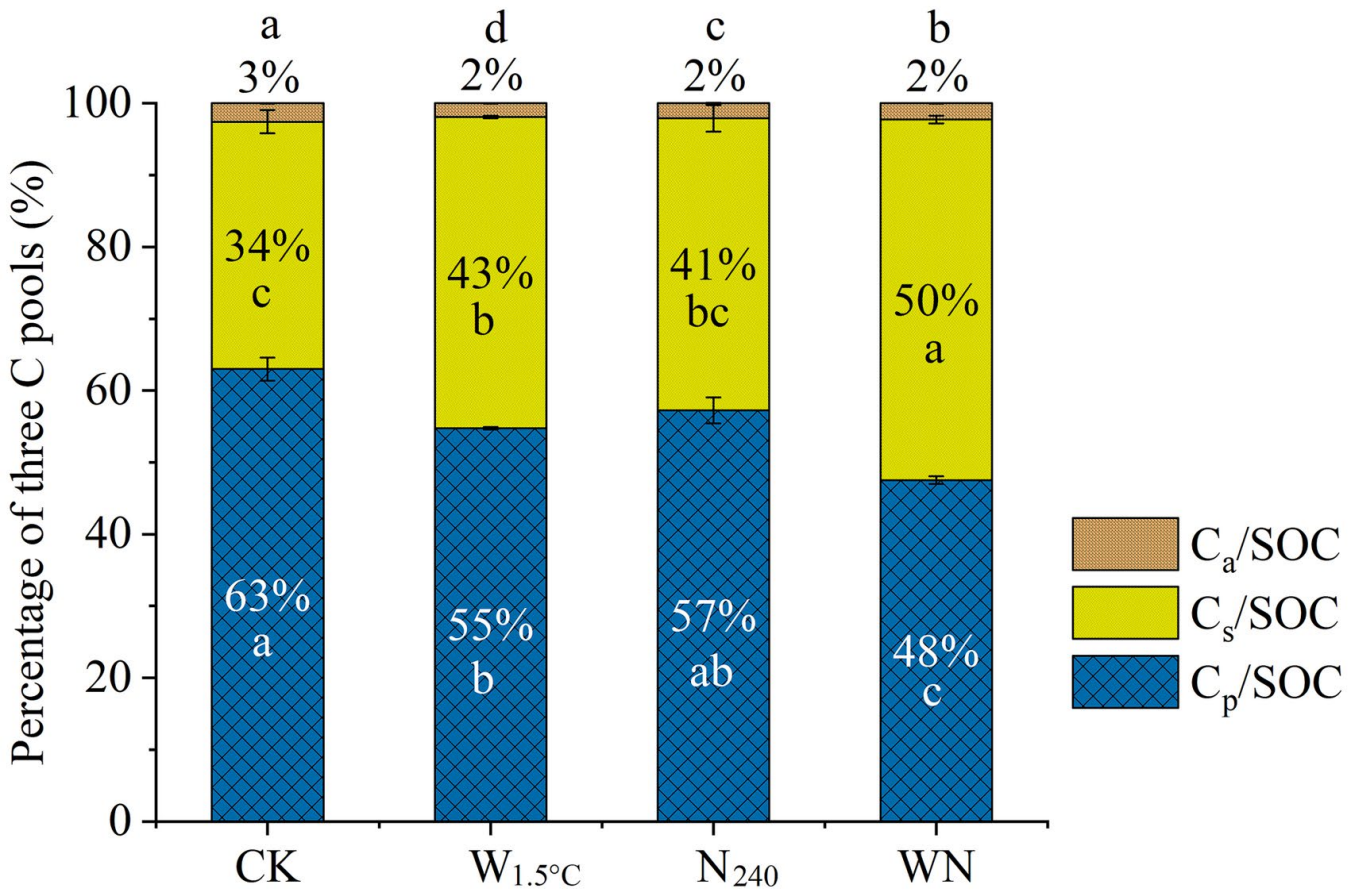
Relative contributions of three carbon pools to total soil organic carbon in soils exposed to 15 years of warming and nitrogen fertilization. Lowercase letters indicate statistically significant differences between treatment groups (*n* = 3, *p* < 0.05).

Warming alone caused a 30% reduction in C_a_ sizes, exceeding the 19% decrease in the N_240_ treatment and the 14% decrease in the WN treatment, relative to the control. Soils exposed to warming plus nitrogen fertilization showed a 26% decrease in C_p_ sizes, surpassing the 18 and 10% decreases observed in the warming-only and N fertilization-only treatments, respectively (Figure 1a and Table 2). As a result, C_a_/SOC decreased by approximately 1% across all treatments. The C_p_/SOC ratio declined by 8, 6, and 15% in the warming, N fertilization, and WN treatments, respectively, compared to the CK (Figure 2). C_s_ sizes in the WN treatment exhibited the highest increase, approximately 41%, surpassing the ~18% increases observed in the sole warming and N fertilization treatments (Table 2). This corresponded to the largest increase in the C_s_/SOC ratio, with a 16% rise in the WN treatment, compared to the increases of approximately 9 and 7% in the warming and N fertilization treatments, respectively, relative to the CK (Figure 2). Compared to the CK, MRT_a_ decreased by approximately 1.9, 0.6, and 1.0 days, while MRT_s_ increased by 220, 151, and 533 days in the warming, N fertilization, and WN treatments, respectively (Figure 1a and Table 2).

**Table 2:** Estimated sizes of three soil carbon pools based on first-order equation models in response to long-term warming and nitrogen fertilization, and the interactive effects of both treatments.

Treatments	C_a_	MRT_a_	C_s_	MRT_s_	C_p_
	mg kg^−1^	Days	g kg^−1^	days	g kg^−1^
CK	198.3 ± 1.5 a	6.7 ± 0.1 a	2.7 ± 0.2 c	863.7 ± 53.0 c	4.9 ± 0.1 a
W_1.5°C_	138.1 ± 1.4 d	3.8 ± 0.0 d	3.1 ± 0.0 b	1,084.1 ± 4.2 b	4.0 ± 0.0 c
N_240_	161.4 ± 2.4 c	6.1 ± 0.1 b	3.1 ± 0.2 b	1,015.0 ± 60.8 bc	4.4 ± 0.1 b
WN	171.3 ± 2.6 b	5.7 ± 0.1 c	3.8 ± 0.1 a	1,396.3 ± 44.6 a	3.6 ± 0.0 d
W_1.5°C_	ns	**	**	**	**
N_240_	**	**	**	**	**
W_1.5°C_ × N_240_	**	**	ns	ns	ns

The reported values are presented as means ± standard errors (SE). Lowercase letters denote significant differences between treatments (*n* = 3, *p* < 0.05). C_a_, C_s_, and C_p_ represent active, stable, and passive carbon pools, respectively. MRT_a_ and MRT_s_ denote the mean residence times of active and stable carbon pools. Asterisks (**) indicate significance at *p* < 0.01, while ns denotes no significance (*p* > 0.05).

### 3.3 Responses of MBC, CUE, and δ^13^C-MBC to straw addition in warming and N-fertilized soils

**Figure 3:**
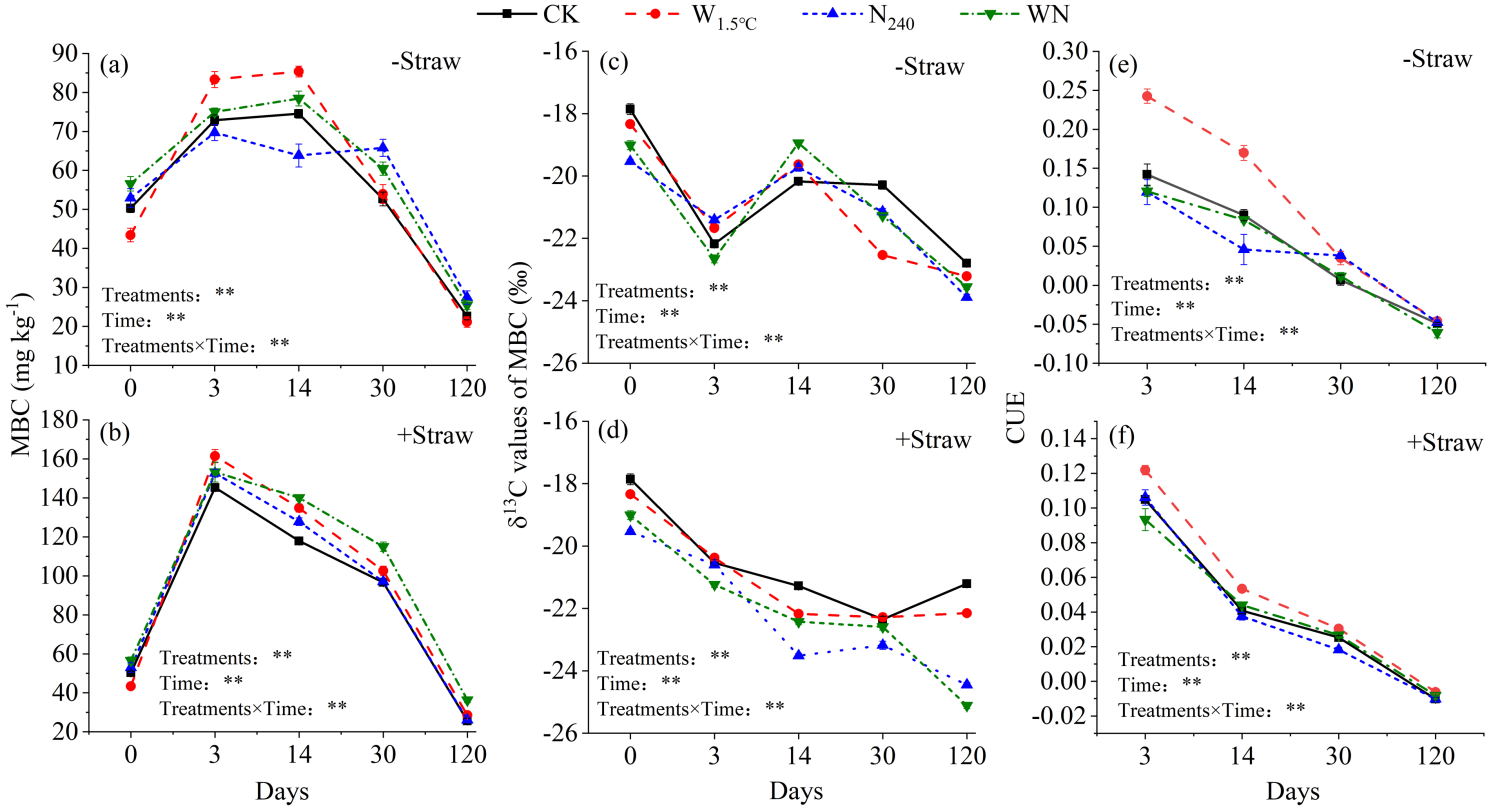
Temporal dynamics of microbial biomass carbon (MBC), corresponding δ^13^C-MBC values, and microbial carbon use efficiency (CUE) in response to air **(a,c,e)** and straw **(b,d,f)** amendments in soils subjected to 15 years of warming and nitrogen fertilization.

MBC, CUE, and δ^13^C-MBC were synchronously influenced by both treatments and incubation time (*p* < 0.01). A total of 13 years of warming declined field soil MBC content by approximately 14%, whereas the combined effects of warming and N fertilization led to a contrasting increase of roughly 13% (Figure 3a). δ^13^C-MBC values exhibited significant depletions under both N fertilization (1.7‰) and WN treatments (1.2‰), as compared to the control (Figure 3c). Field soils subjected to sole warming exhibited a remarkable increase in CUE, ranging from 67 to 82% higher than in soils under N fertilization, combined warming and N fertilization, and the untreated control (Figure 3e). Compared to the CK, δ^13^C-MBC values under sole warming or N fertilization were more positive (0.44‰ to 0.77‰ higher) on the 3rd and 14th days but became relatively negative by the 30th and 120th days (0.86‰ to 2.25‰ lower). In the WN treatment, δ^13^C-MBC values remained negatively shifted by 0.47–0.99‰ relative to CK on the 3rd, 30th, and 120th days of incubation, except for a brief positive excursion (1.23‰) on the 14th day (Figure 3c).

MBC content in straw-amended soils reached its peak on the third day of incubation (Figure 3b). Compared to the untreated control, the peak MBC values increased by approximately 19% in sole warming soils, exceeding the 5% increase observed in the N fertilization and WN treatments (Figure 3b). In soils subjected to combined warming and N fertilization, MBC content exceeded that of the CK, warming-alone, and N-fertilization-alone treatments by approximately 42% on the 120th day of incubation (Figure 3b). In straw-amended soils, warming alone led to a 19% increase in CUE, whereas the WN treatment resulted in a decrease of approximately 10% in CUE compared to the N fertilization and CK treatments (Figure 3f). In soils exposed to warming alone or combined with N fertilization, δ^13^C-MBC values were 0.89–3.90‰ more negative compared to the CK on the 14th and 120th days of incubation. Nitrogen fertilization alone caused more negative δ^13^C-MBC values than the CK, ranging from 0.83‰ to 3.13‰ between the 3rd and 120th days of incubation (Figure 3b).

### 3.4 PE in response to warming and N fertilization during the incubation period

**Figure 4:**
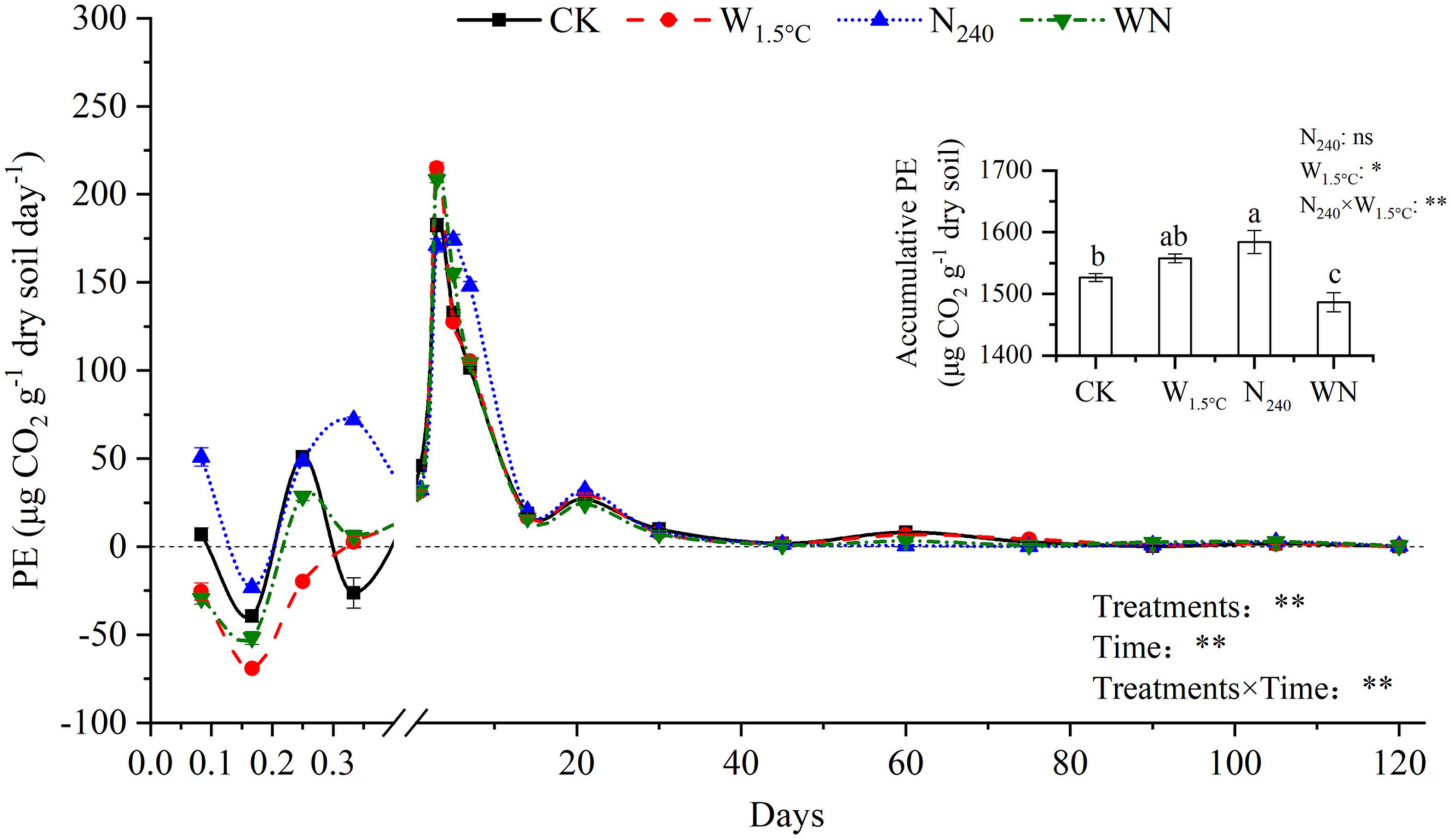
Priming effects (PEs) mediated by 15 years of warming and nitrogen fertilization during incubation. Lowercase letters denote statistically significant differences between treatments (*n* = 3, *p* < 0.05).

The magnitude of PEs was synchronously influenced by both treatments and incubation time (*p* < 0.01). The PE was generally negative during the first 6 h, reached a peak on the third day of incubation, and subsequently declined thereafter (Figure 4). Warming alone and the combined warming plus N fertilization treatment resulted in PE levels that were 0.4–5.2 times lower than the untreated control during the initial 6 h of incubation. The peak of the PE was 18 and 14% higher in soils subjected to warming alone and the combined warming plus N fertilization treatment, respectively, compared to the CK. Between the 14th and 75th days of incubation, the combined warming plus N fertilization treatment caused a more pronounced decrease in the PE than the warming-only treatment, ranging from 10 to 68% *versus* 4–17% lower than the CK. Ultimately, combining warming with N fertilization led to a significant reduction of 3% in cumulative PEs compared to the untreated control, whereas warming alone and N fertilization alone increased PEs by approximately 2 and 4%, respectively, relative to the CK (Figure 4).

### 3.5 Relationship analysis

**Figure 5:**
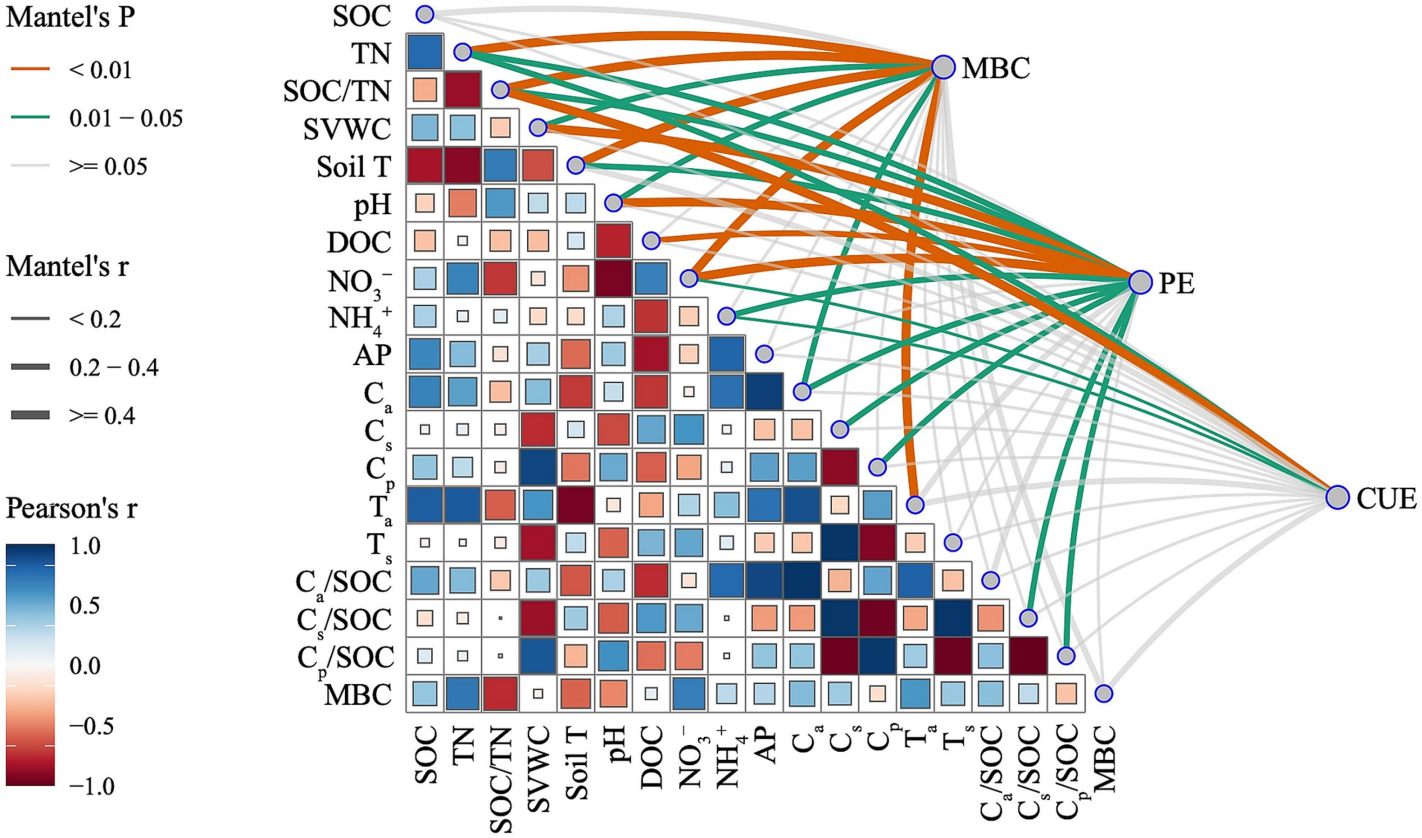
Mantel test and Pearson correlation analyses exploring the relationships between priming effects (PE), microbial biomass carbon (MBC), and microbial carbon use efficiency (CUE) induced by straw additions and ambient soil physicochemical properties (*n* = 12). Line color and size represent the *p* and *r* values of the Mantel test, respectively. Square color and size depict the *p* and *r* values of Pearson correlation analysis, respectively.

Pearson’s correlation analysis showed that C_a_, T_a_, and C_a_/SOC were positively associated with AP but negatively correlated with soil T and DOC. C_s_, T_s_, and C_s_/SOC were negatively associated with SVWC (*p* < 0.05, Figure 5). The Mantel test suggested that straw-induced MBC during incubation was significantly related to TN, SOC/TN, soil T, and T_a_ (*p* < 0.01), as well as to C_a_, SVWC, and pH (*p* < 0.05). The PE dynamic during incubation was significantly related to SVWC, pH, DOC, and NO_3_^−^ (*p* < 0.01), as well as to TN, SOC/TN, soil T, NO_4_^+^, C_a_, C_s_, C_p_, C_s_/SOC, and C_p_/SOC (*p* < 0.05). CUE was sensitive to the SOC/TN ratio (*p* < 0.01) and also showed significant associations with TN, NO_3_^−^, and NH_4_^+^ (*p* < 0.05, Figure 5).

**Figure 6:**
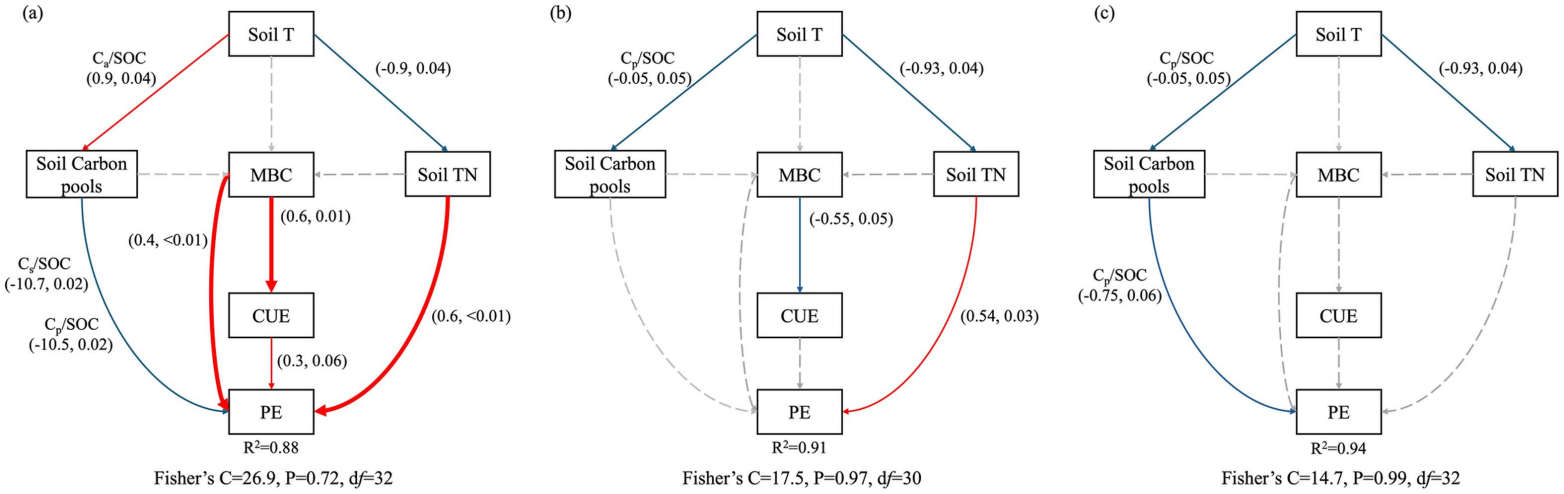
The direct and indirect interactions among ambient soil carbon pools, nutrient contents, initial microbial biomass, and CUE on priming effects (PEs) were elucidated using structural equation models (SEMs) across three distinct incubation periods: **(a)** The initial 2 h, **(b)** up to 14 days, and **(c)** 30 days (*n* = 12). Soil T and TN represent soil temperature and total nitrogen content at a depth of 10 cm, respectively. C_a_/SOC, C_s_/SOC, and C_p_/SOC denote the ratios of labile, stable, and passive carbon pool sizes to total soil organic carbon. MBC and CUE represent initial microbial biomass carbon and carbon use efficiency. Blue solid lines denote significant negative effects, and red solid lines signify significant positive correlations (*p* < 0.05). Gray dotted lines denote the lack of significant correlations (*p* > 0.05).

The SEM effectively delineated the influence of ambient soil physical properties (Soil T), nutrient contents (encompassing soil carbon pools component and TN), and initial microbial properties (MBC and CUE) on PEs across three pivotal incubation time points: the 3rd, 14th, and 30th days. The model demonstrated satisfactory reliability (Fisher’s C *p* > 0.05), explaining 88, 91, and 94% of the variability in PEs at the second hour, 14th day, and 30th day, respectively (Figures 6a–c). Within the first 2 h of incubation, soil T positively modulated the C_a_/SOC ratio while negatively affecting PEs through inverse correlations with the C_s_/SOC and C_p_/SOC ratios. In addition, initial soil MBC and CUE exerted both direct and indirect effects on PEs (Figure 6a). On the 14th day of incubation, soil T was negatively correlated with TN, with a concomitant positive impact on PEs (Figure 6b). Ultimately, on the 30th day of incubation, soil T exhibited a negative correlation with the C_p_/SOC ratio, which directly translated into a negative influence on PEs (Figure 6c).

## 4 Discussion

### 4.1 Changes in carbon pools in ambient soils subjected to long-term warming and N fertilization

Contrary to our first hypothesis, 13-year warming significantly destabilized soil carbon pools, resulting in a 26% reduction in C_p_ coupled with a 41% increase in C_s_ size (Table 2 and Figure 2). This divergence arises from synergistic mediation by warming-induced moisture stress and nutrient limitation. Long-term warming amplified microbial nitrogen demands, which might drive the microbial transformation of C_p_ into C_s_ pools. These patterns were supported by three lines of evidence: (1) Extended MRT of C_s_ pools confirmed the preferential stabilization of slow-cycling fractions derived from C_p_ degradation (Figure 1a and Table 2). (2) Microbial CUE exhibited a strong negative correlation with TN (Figures 3e, 5), indicating that nutrient-limited microbes prioritized growth efficiency while mining passive substrates to alleviate N deficits (Shi et al., 2023). (3) C_a_ size decreased with TN and SVWC, while C_s_ size increased with NO_3_^−^ (Tables 1, 2 and (Figure 5). Consistently, results from an 8-year warming experiment also supported this pattern, showing that oxidase activity per unit MBC increased relative to the control (Zhang C. et al., 2021), facilitating C_p_ decomposition. This result aligns with observations from temperate forests under 15 years of warming, where the dominance of passive carbon-degrading microbes triggered secondary CO_2_ emission peaks (Melillo et al., 2017; Metcalfe, 2017).

Consistent with our second hypothesis, the combined treatment (WN) caused a more pronounced reduction in C_p_ size, accompanied by a greater accumulation of C_s_ size, compared to soils subjected to either warming or N fertilization alone (Table 2 and Figure 2). The higher MRT of C_s_ in the WN treatment compared to either treatment alone implies a faster transformation of C_p_ into C_s_ (Figure 1a and Table 2). Despite baseline P fertilization, the WN treatment exhibited the smallest decline in available P (Table 1), indicating intensified P mining from passive carbon. These findings are in line with Shi et al. (2023) and Gong et al. (2020), supporting the idea that nutrient elements are released to meet microbial energy demands. However, the WN treatment exhibited a microbial CUE similar to that of the CK (Figure 3e). Furthermore, results in the 8th year revealed that the WN treatment caused the highest increase in microbial biomass and C-, N-, and P-acquiring hydrolase activities (Zhang C. et al., 2021). This indicates that microorganisms in the WN treatment allocated resources to satisfy both elemental limitations and growth demands, in contrast to those in the warming-only treatment. These findings imply that soil carbon in farmland is susceptible to future climate warming. In addition, tillage prior to sowing can break apart soil aggregates, thereby disrupting the physical protection of carbon and increasing its accessibility to microbes (Lehmann et al., 2020). This process might ultimately accelerate the decomposition of the passive carbon pool.

Long-term N fertilization also transformed passive carbon pools into slow carbon pools (Table 2 and Figure 2). Contrary to global meta-analyses, long-term N fertilization-induced acidification preserved soil organic carbon derived from litter, primarily within the active carbon fractions (Tian and Niu, 2015; Ye et al., 2018; Chen et al., 2023). This pattern resulted from microbial anabolism exceeding catabolism in N-fertilized soils, as evidenced by: (1) increased MBC and depleted δ^13^C values in both SOC and MBC in N-fertilized soils compared to the CK (Table 1 and Figure 3c), indicating preferential assimilation of passive substrates. (2) Increased soil amino sugar contents under N fertilization (Supplementary Figure S1), confirming the contribution of microbial-derived carbon to C_s_ pools. These findings underscore the role of microbial pumping in driving the transformation of C_p_ into C_s_ pools (Liang et al., 2017). In addition, reduced alkalinity (Table 1) and tillage might increase the accessibility of C_p_ pools, further contributing to this transformation.

### 4.2 Priming effects in response to long-term warming and N fertilization

Long-term warming and N fertilization alone increased cumulative PE intensity, while their combined effects exhibited a decrease in cumulative PE intensity (Figure 4). These findings contrast with the general pattern in which lower microbial CUE leads to higher PE intensity (Mau et al., 2018). This discrepancy may be attributed to the control exerted by microbial CUE and biomass on the PE during the early stages of incubation (Figure 6a). In warming-alone soils, higher microbial CUE drove lower but still negative PEs during the first 6 h of incubation (Figures 3e, 4). Oligotrophic soils favor microorganisms with higher CUE, which generally results in lower PEs (Chen et al., 2014; Li et al., 2023). However, in warming soils, the highest PE values on the 3rd day offset the decreases during the first h and the subsequent stages of incubation (14 ~ 120 days), ultimately increased the cumulative PE (Figure 4). SEM showed that TN and the C_p_/SOC ratio controlled PE intensity on the 14th and 30th days, respectively (Figures 5, 6b,c). The decline in cumulative PEs observed under the combined treatment (Figure 5) was attributed to the decrease in C_p_ size (Table 2 and Figure 2). This laboratory-level evidence underscores the critical role of soil carbon stability in regulating both PE direction and intensity.

The composition of the soil carbon pool influenced distinct microbial substrate allocation strategies during straw incorporation. In the WN treatment, MBC increased, with δ^13^C-MBC values converging toward straw-derived carbon over the course of incubation (Figures 3b,d). In the WN treatment, soil amino sugar content also increased (Supplementary Figure S1). In contrast, straw-derived carbon was primarily catabolized to meet energy demands in warming-only soils, with MBC and δ^13^C-MBC values returning to levels similar to the CK (Figures 3b,d). These findings highlight that microbial anabolism exceeded catabolism in the combined treatment (Liang et al., 2017; Zhu et al., 2020). These shifts in microbial metabolism might be driven by nutrient stoichiometric constraints. Microbial competition for substrates likely underlies the observed variation in the priming effect.

### 4.3 Limitations and implications

Our findings underscore that the combined effects of long-term warming and nitrogen additions accelerated the decomposition of passive carbon and increased the accumulation of slow carbon relative to the individual treatments. Shifts in microbial nutrient demands, such as higher nitrogen demand under warming alone and higher phosphorus demand under the combined treatment, altered microbial metabolism, ultimately accelerating the destabilization of soil carbon pools. Our study was conducted in a semi-arid, alkaline cropland system with a history of high nitrogen inputs. The 3% decrease in soil moisture induced by warming is unlikely to be the primary driver of carbon decomposition. In addition, nitrogen additions partially alleviated soil alkalinity, although the change in soil pH was not statistically significant. Tillage prior to sowing increased microbial accessibility to substrates, further facilitating the decomposition of passive carbon in the combined treatment. In semi-arid croplands of the North China Plain, reducing nitrogen fertilization may help stabilize soil carbon under future global warming.

## 5 Conclusion

Based on a 13-year field experiment in North China Plain farmland, this study suggests that both warming and nitrogen fertilization individually accelerated the transformation of passive carbon into slow carbon pools. The combined treatment further amplified these trends, resulting in greater depletion of passive carbon and increased accumulation of slow carbon compared to either treatment alone. Straw incorporation led to higher microbial biomass carbon and more negative δ^13^C-MBC values in soils subjected to combined warming and nitrogen fertilization by the 120th day of incubation, relative to the control and individual treatments. These findings imply that microbial anabolism exceeded catabolism under the combined treatment. In soils subjected to warming alone, microbial biomass peaked on the third day of incubation and returned to control levels by the 120th day following straw incorporation. These results suggest that long-term warming promoted higher catabolic activity compared to anabolism. Both warming and nitrogen fertilization individually enhanced the cumulative PE induced by straw incorporation, while their combined treatment reduced it compared to the control. This reduction in the cumulative PE was associated with lower microbial carbon use efficiency. Collectively, our results suggest that the combined effects of long-term warming and nitrogen fertilization likely accelerated the decomposition of passive carbon and stimulated greater microbial anabolism in semi-arid, alkaline farmland. This metabolic shift may represent a key mechanism driving soil carbon cycling in farmland under future climate warming.

## Data Availability

The original contributions presented in the study are included in the article/Supplementary material, further inquiries can be directed to the corresponding author.
