# Supplementary material for: Synergistic effects of 13-year warming and nitrogen fertilization accelerating soil carbon destabilization in North China Plain farmland
Source: Front Microbiol. 2026 Apr 16;17:1775179. doi: 10.3389/fmicb.2026.1775179 (PMC13128580; doi:10.3389/fmicb.2026.1775179)
Supplement: Supplementary file 1 [file Table_1.DOCX]

# **Supplemental material:**

**Figs. S1** Modulations of total Amino Sugar contents in response to warming and nitrogen fertilization over the eight-year experimental period. Small letters denote statistically significant differences between treatments (n=6, p<0.05), as assessed using One-way ANOVA, followed by Turkey post hoc test.
